# Association between air temperature and emergency admission for esophagogastric variceal bleeding: a case-crossover study in Beijing, China

**DOI:** 10.1186/s12876-023-02683-w

**Published:** 2023-02-25

**Authors:** Jianhong Chen, Ziting Wu, Hui Gao, Li Li, Yanling Wang, Jingjing Han, Chuan Zhang, Pengpeng Ding, Jing Wu

**Affiliations:** 1 Department of Gastroenterology, Beijing Tongren Hospital, Capital Medical University, Beijing, China; 2 School of Public Health, Peking University, Beijing, China; 3 National Climate Center, China Meteorological Administration, Beijing, China; 4 Department of Gastroenterology, Beijing Shijitan Hospital, Capital Medical University, Beijing, China; 5 Cirrhosis Diagnosis and Treatment Center, Fifth Medical Center of PLA General Hospital, Beijing, China; 6 Department of Gastroenterology, Beijing Friendship Hospital, Capital Medical University; National Clinical Research Center for Digestive Diseases; Beijing Digestive Disease Center, Beijing Key Laboratory for Precancerous Lesion of Digestive Diseases, Beijing, China

**Keywords:** Temperature, Liver cirrhosis, Esophageal and gastric varices, Cross-over studies

## Abstract

**Background and aims:**

Studies concerning the impact of air temperature on esophagogastric variceal bleeding (EGVB) have yielded conflicting results. Our study aimed to evaluate the correlation between air temperature and EGVB.

**Methods:**

A time-stratified case-crossover study design was performed. Patients received emergency gastroscopic hemostasis for upper gastrointestinal bleeding between Jan 1, 2014, and Dec 31, 2018 in the Fifth Medical Center of PLA General Hospital were enrolled. Conditional logistic regression analysis was applied to determine the association between air temperature and EGVB for different lag structures.

**Results:**

A total of 4204 cirrhotic patients diagnosed with EGVB and received emergency gastroscopic hemostasis were enrolled. The mean number of daily EGVB cases peaked in October (2.65 ± 1.69) and fell to the lowest level in July (1.86 ± 1.38), and was 2.38 ± 1.58 in spring, 2.00 ± 1.46 in summer, 2.37 ± 1.58 in autumn, and 2.45 ± 1.58 in winter, respectively (P < 0.0001). In conditional logistic regression analysis, no significant correlations between air temperature and EGVB were observed and no significant difference were found when stratified by age, sex, etiology, liver cancer status, and grade of varices.

**Conclusion:**

Emergency admission for EGVB showed significant monthly and seasonal fluctuations, while in conditional logistic regression analysis, no association between minimum temperature and emergency admission for EGVB were observed.

## Introduction

Esophagogastric variceal bleeding (EGVB) is the most life-threatening complication of cirrhosis and occurs in 5–15% of Esophagogastric varices (EV) patients annually [1]. EGVB occurs in one third of patients with EV and causes 70% of upper gastrointestinal bleeding episodes in cirrhotic patients [2]. The commonly identified risk factors for EGVB include a higher hepatic vein pressure gradient, larger EV size, the presence of red wale marks, portal vein thrombosis, alcohol consumption, poor renal function, severe cirrhosis and hepatocellular carcinoma (HCC) [3].

Meteorological factors are closely related to human health, and the evidence is generally clear that air temperature is associated with higher incidence and mortality in patients with cardiorespiratory and cerebrovascular diseases [4–7]. Several previous studies have analyzed the effect of seasonal variation and air temperature on the incidence of EGVB, the conclusions were inconsistent and controversial [3, 8–12]. Low temperature exposure may increase cardiac output and result in an increase in intrahepatic vascular resistance in animal studies [10, 11]. Increases in blood pressure, peripheral resistance, the activation of the renin–angiotensin–aldosterone system and reductions of perspiration have also been reported in individuals exposed to lower temperature [10, 11]. These physical responses may lead to a higher hepatic vein pressure gradient and cause EGVB. The objective of this study was to elucidate the association between air temperature and emergency admission for EGVB in Beijing using a time-stratified case-crossover design and conditional logistic regression analysis.

## Methods

### Study design

**Fig. 1:**
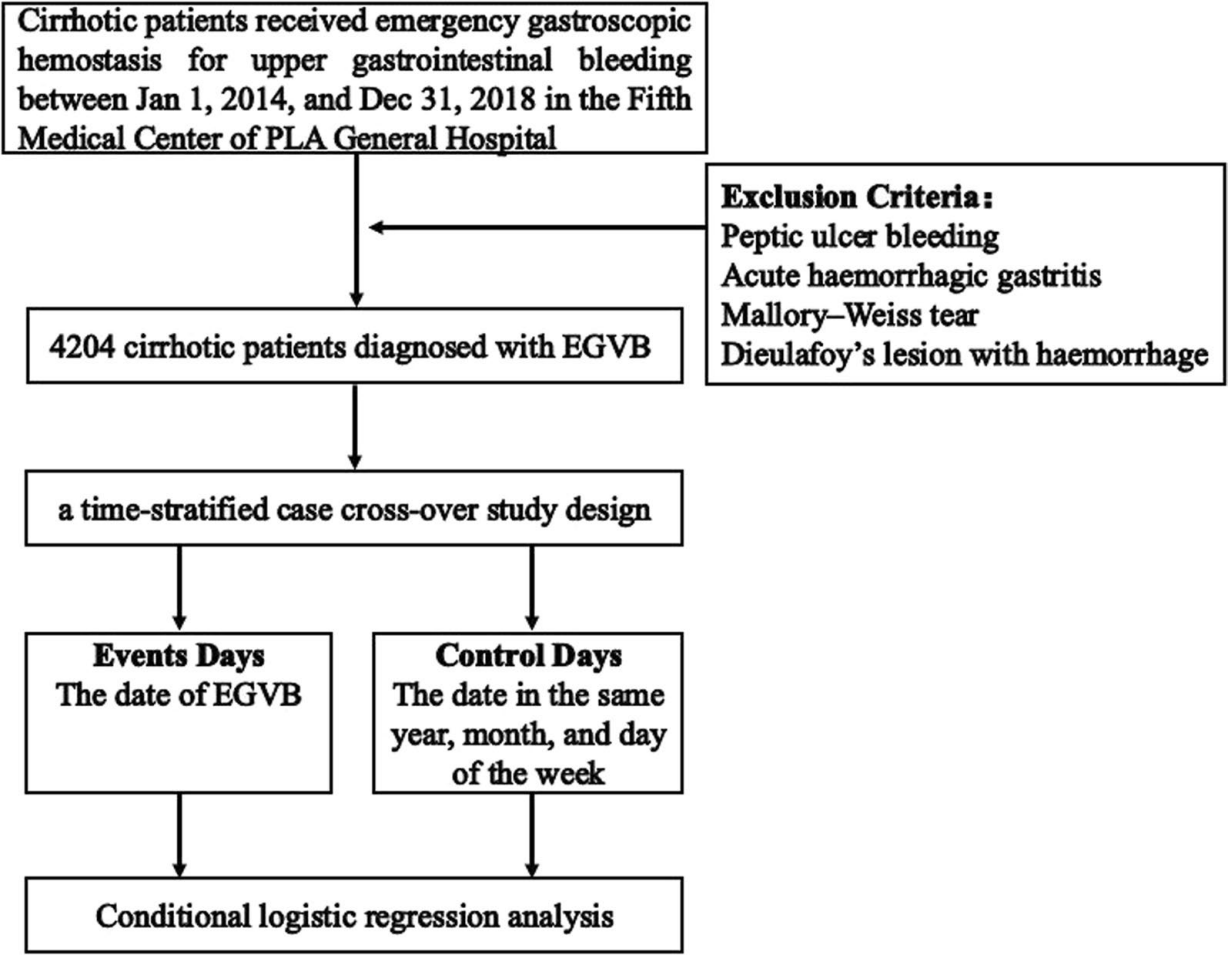
Flow chart of study design

Our study used a time-stratified case-crossover study which had been widely used to examine short-term environmental exposure factors on acute cardiovascular and cerebrovascular events [13–15]. We analyzed the association between the air temperature and EGVB through comparing the emergency admission for EGVB on the event days and control days. A time-stratified method was used to select control days which were in the same calendar year, month, and day of the week to match with specific time-varying confounders and ensure unbiased conditional logistic regression estimates (Fig. 1). Exposure level during the case period was defined as minimum temperature on the event day (lag0) or 1–7 days preceding event day (lag 1–7).

### Study population and meteorological data

We collected clinical characteristics (including sex, age, date of admission, clinical diagnosis and endoscopic diagnosis) of patients received emergency gastroscopic hemostasis for upper gastrointestinal bleeding between Jan 1, 2014, and Dec 31, 2018 in the Fifth Medical Center of PLA General Hospital. All diseases were coded by International Classifications of Diseases version 10 (ICD-10). Patients with a diagnosis of EGVB (ICD-10-K74.609 + and I85.001) treated with endoscopic varices ligation, endoscopic injection sclerotherapy, histoacryl injection, and Sengstaken-Blakemore tube were included. We excluded patients with concomitant aetiologies of upper gastrointestinal bleeding including peptic ulcer bleeding (ICD-10-K27.253, K27.451), acute haemorrhagic gastritis and duodenitis (ICD-10-K29.001, K29.051), Mallory–Weiss tear (ICD-10-K22.601), Dieulafoy’s lesion with haemorrhage (ICD-10-K25.051). Meteorological data on daily minimum temperature, relative humidity (RH%) and atmospheric pressure (AP) between Jan 1, 2014, and Dec 31, 2018 were obtained from the China meteorological administration.

### Statistical analyses

Microsoft Excel (Microsoft, Redmond, Washington, USA) was used for data collection and analysis. Data were expressed as mean ± standard deviation or count number. General and clinical characteristics of all enrolled cases were described, and ANOVA methods were applied to assess the seasonal and monthly variations of daily emergency admission for EGVB.

We then used conditional logistic regression to examine association between air temperature and emergency admission for EGVB for each lag day. Distributed lag non-linear models (DLNM) with three degrees of freedom in the natural cubic splines and a maximum lag of 7 days were used to adjust for the delayed and non-linear effects of temperature. To control for the effects of other meteorological factors on EGVB, we added RH% and AP to the models. The effects of public holidays were not included in the models for the “Green Channel” of emergency medical service for EGVB. We calculated the percentage changes on the risks of EGVB per interquartile range (IQR) increase in minimum temperature on different single lag day (lag0-lag7). The associations with mean minimum temperature (lag01-lag07) on different lag days were also calculated to avoid underestimating the effect of the minimum temperature measured by single lag day model. We analyzed the exposure response association between minimum temperature and emergency admission for EGVB in the event day (lag 0) and different lag days [16]. We stratified the enrolled patients by age, sex, etiology of liver disease, HCC status and grade of varices to analyze the effects of minimum temperature on emergency admission for EGVB.

We carried out statistical analysis with R version 3.6.1 and SPSS version 20.0. All statistical tests were two-sided and P < 0.05 was considered statistically significant.

## Results

A total of 4204 cirrhotic patients diagnosed with EGVB and received emergency gastroscopic hemostasis between Jan 1, 2014, and Dec 31, 2018 in the Fifth Medical Center of PLA General Hospital were enrolled in this study. The mean age was 53.32 ± 11.46 years, 3155 (75.1%) patients were male, 936 (22.3%) patients were diagnosed with liver carcinoma. Main aetiologies of cirrhosis in these patients were HBV infection (62.2%, n = 2615), alcohol liver disease (12.4%, n = 522), HCV infection (8.9%, n = 375), primary biliary cirrhosis (5.1%, n = 212), autoimmune hepatitis (3.7%, n = 155), and drug induced liver injury (1.6%, n = 67), and approximately 5.5% (n = 233) were cryptogenic cirrhosis. 2820 patients were diagnosed as esophageal variceal bleeding, 777 patients were diagnosed as gastric variceal bleeding, 25 patients were diagnosed as isolated gastric variceal bleeding, and the others couldn’t find the definite location of hemorrhage.

**Fig. 2:**
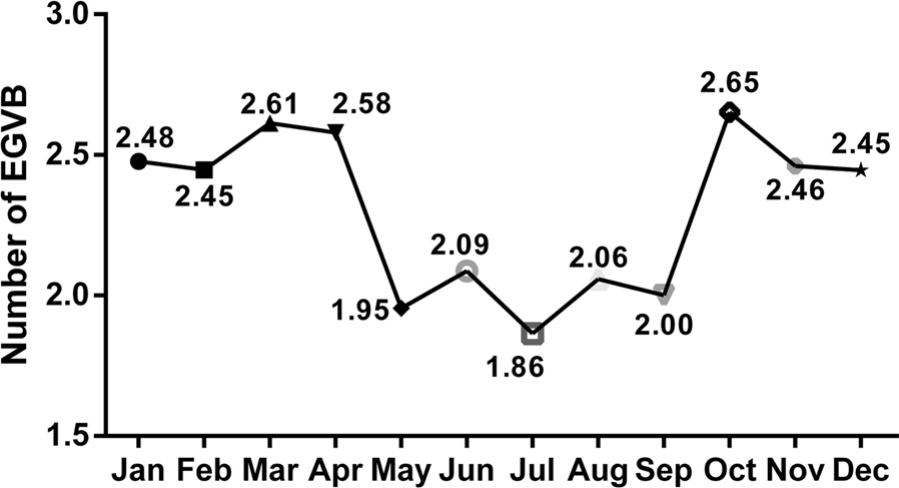
Monthly variation of the mean number of daily emergency admission for EGVB

**Fig. 3:**
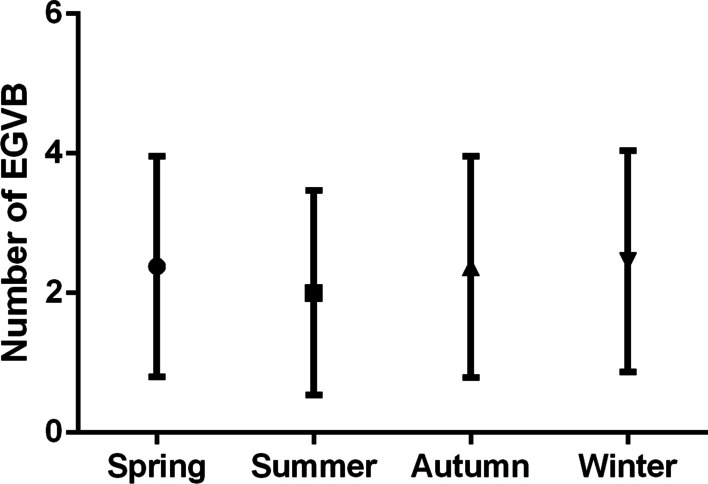
Seasonal variation of the mean number of daily emergency admission for EGVB

The mean number of daily emergency admission for EGVB showed significant monthly and seasonal fluctuations (*P* < 0.0001 and *P* < 0.0001). In Fig. 2, the mean number of daily cases occurred in the colder months were more than that in warmer months. The highest mean number of daily cases occurred in October (2.65 ± 1.69) and the lowest occurred in July (1.86 ± 1.38) (Fig. 2). The mean number of daily emergency admission for EGVB was 2.38 ± 1.58 in spring, 2.00 ± 1.46 in summer, 2.37 ± 1.58 in autumn, and 2.45 ± 1.58 in winter, respectively (Fig. 3).

**Fig. 4:**
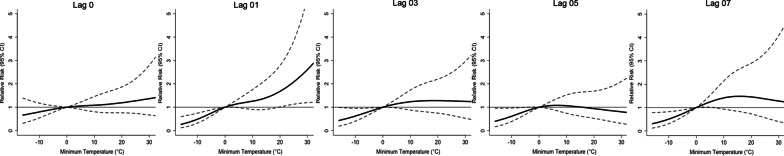
DLNM analysis for associations between minimum temperature and the risks of emergency admission for EGVB adjusted for RH% and AP

**Fig. 5:**
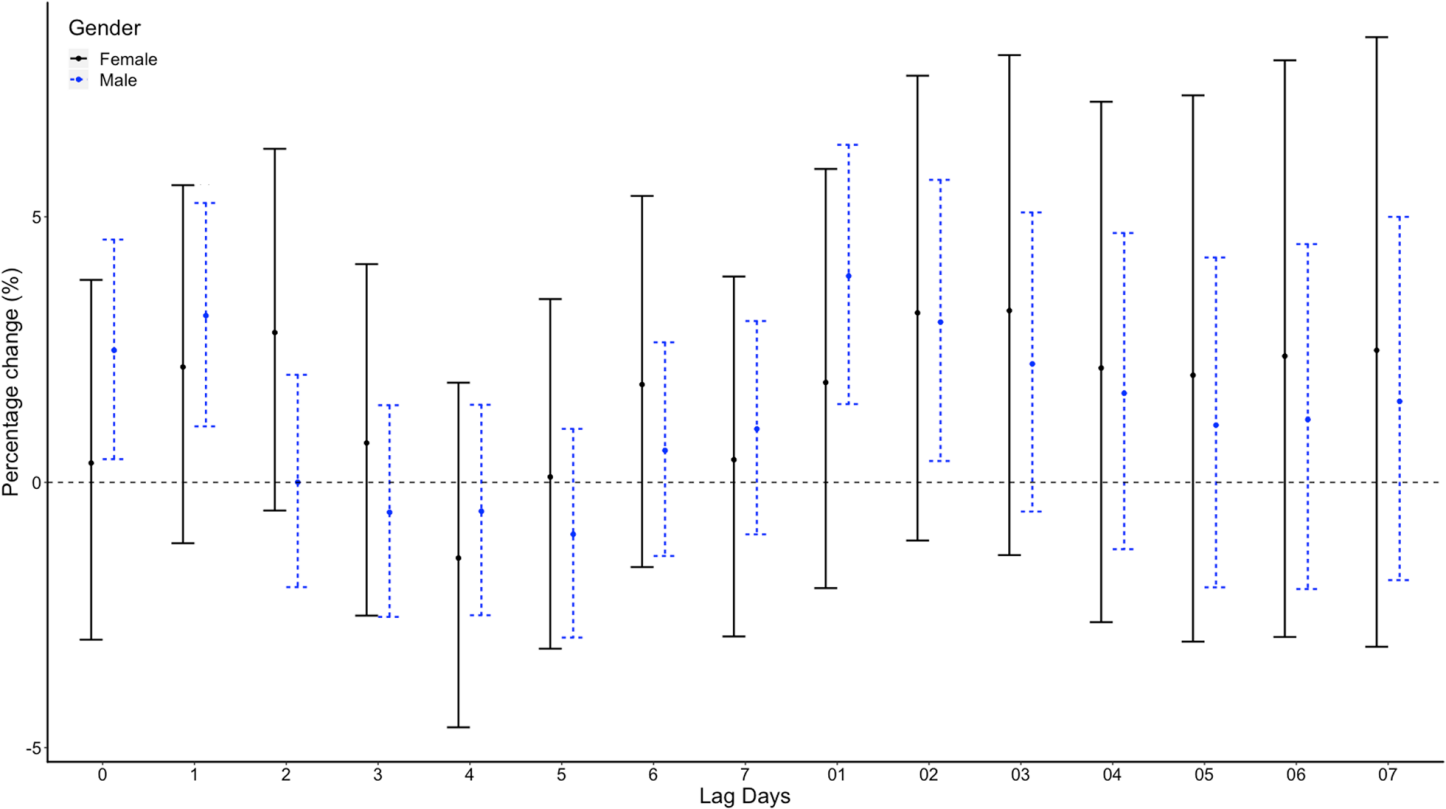
Percentage changes with 95% CI on the risks of EGVB per IQR increase in minimum temperature for different lag structures stratified by sex

**Fig. 6:**
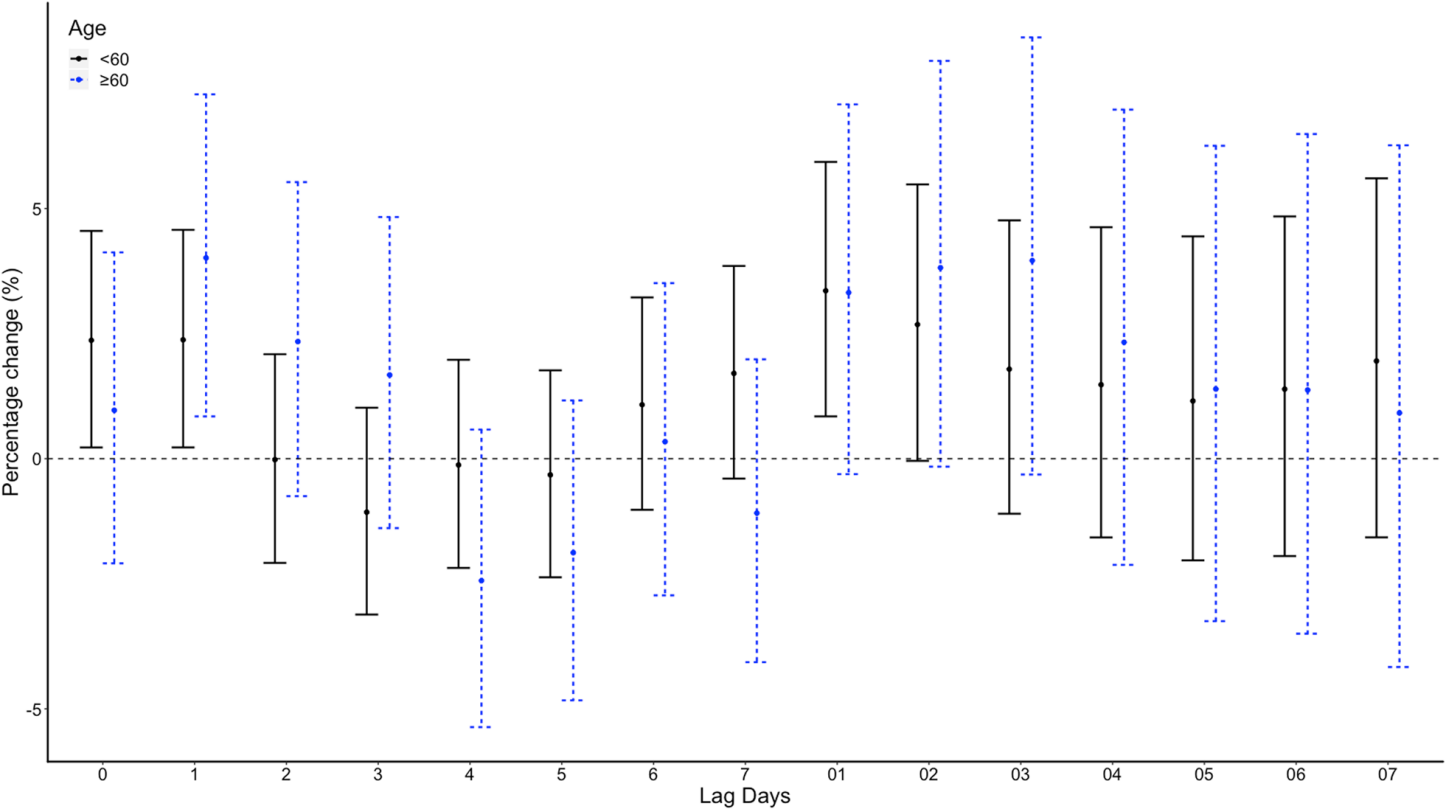
Percentage changes with 95% CI on the risks of EGVB per IQR increase in minimum temperature for different lag structures stratified by age

**Fig. 7:**
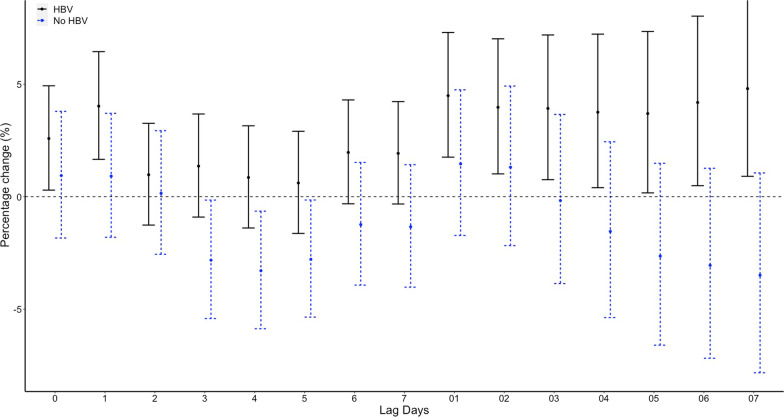
Percentage changes with 95% CI on the risks of EGVB per IQR increase in minimum temperature for different lag structures stratified by etiology

**Fig. 8:**
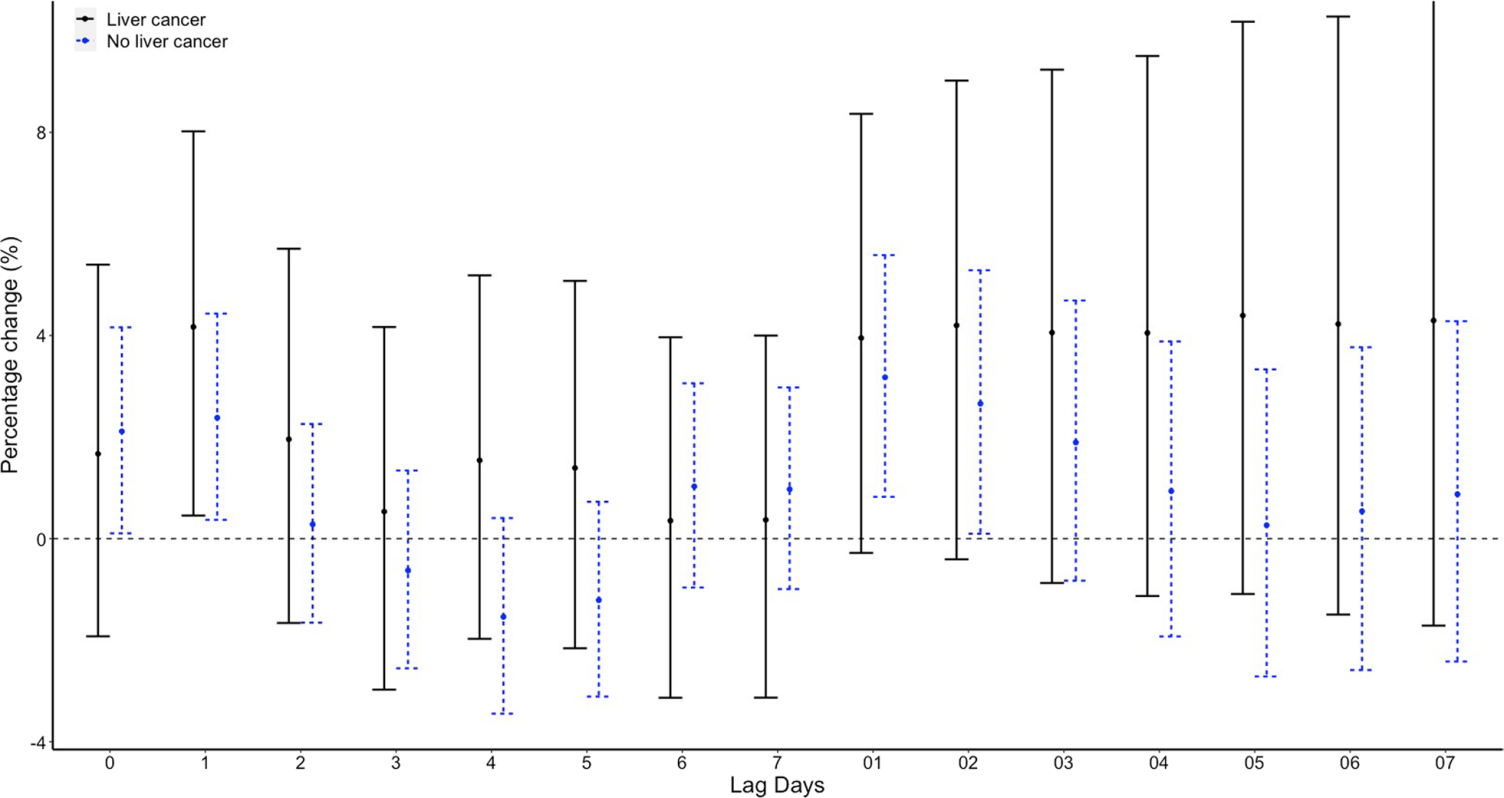
Percentage changes with 95% CI on the risks of EGVB per IQR increase in minimum temperature for different lag structures stratified by liver cancer status

**Fig. 9:**
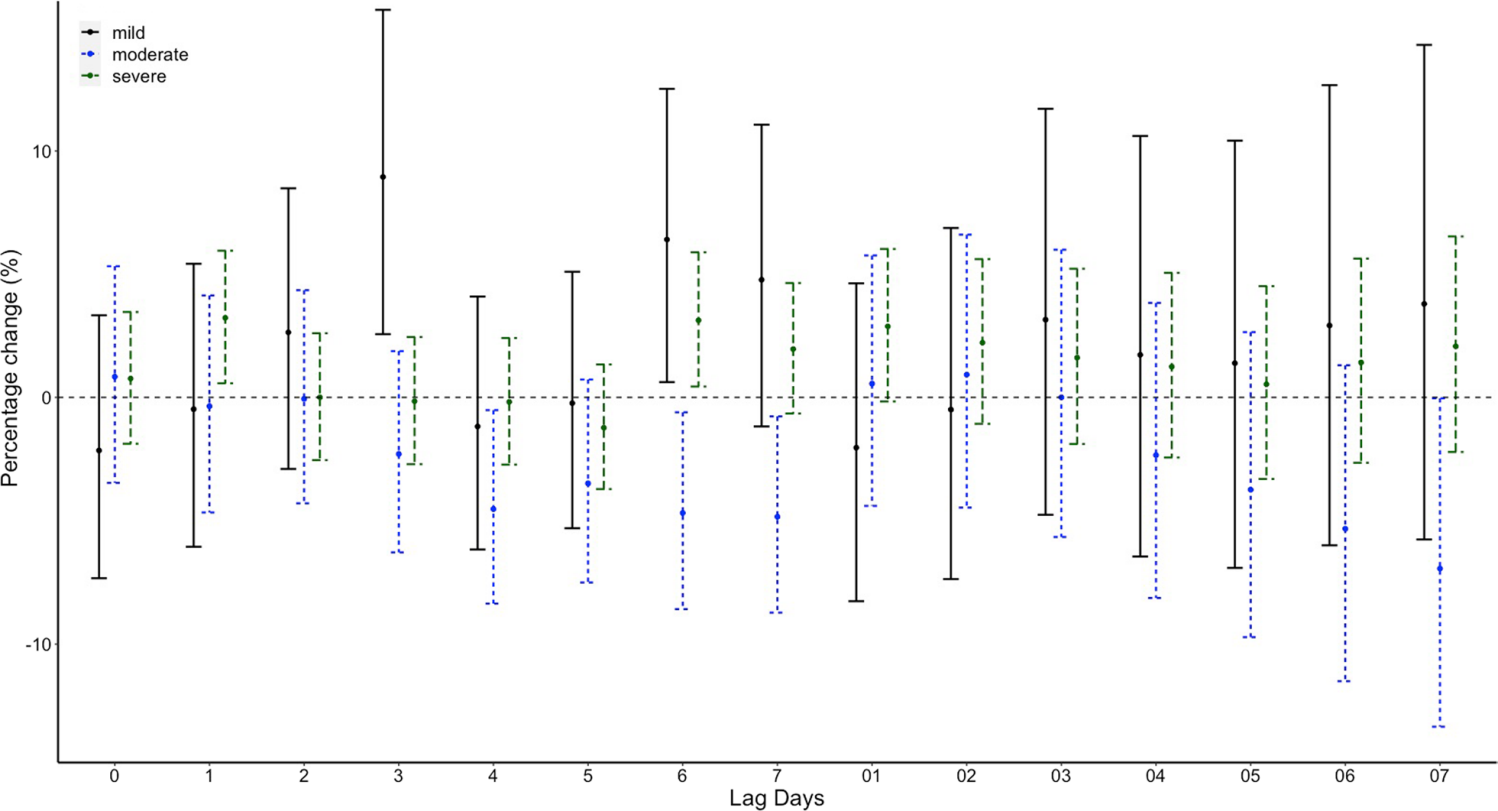
Percentage changes with 95% CI on the risks of EGVB per IQR increase in minimum temperature for different lag structures stratified by grade of varices

While in conditional logistic regression analysis, no correlations between the minimum temperature and emergency admission for EGVB were observed on lag 0. RRs of emergency admission for EGVB were 1.26 (95% CI 0.83–1.9, p = 0.275) for a temperature of > − 10 and <  = 0 compared with a temperature <  = − 10, 1.34 (95% CI 0.85–2.09, *p* = 0.208) for a temperature of > 0 and <  = 10, 1.32 (95% CI 0.81–2.15, *p* = 0.263) for a temperature of > 10 and <  = 20, and 1.35 (95% CI 0.81–2.27, *p* = 0.253) for a temperature of > 20 after adjusted for RH% and AP (Table 1). No significant associations were found on different lag structures in DLNM analysis (Fig. 4). When stratified by sex, age, etiology of liver disease, HCC status, grade of varices, no significant difference were found (Fig. 5, Fig. 6, Fig. 7, Fig. 8, and Fig. 9).

**Table 1:** Associations between the minimum temperature and emergency admission for EGVB in conditional logistic regression analysis before and after adjusted for RH% and AP on lag 0

Groups	Cases (%)	Controls (%)	RRs (95% CI)		*P* value	
			Unadjusted	Adjusted	Unadjusted	Adjusted
< = − 10 °C	34 (0.81)	191 (1.03)	referent	referent	referent	referent
> − 10 °C and < = 0 °C	1411 (33.56)	6174 (33.42)	1.36(0.91–2.02)	1.26 (0.83–1.9)	0.131	0.275
> 0 °Cand < = 10 °C	898 (21.36)	3920 (21.22)	1.44(0.95–2.19)	1.34 (0.85–2.09)	0.088	0.208
> 10 °C and < = 20 °C	1144 (27.21)	4999 (27.06)	1.45(0.94–2.26)	1.32 (0.81–2.15)	0.096	0.263
> 20 °C	717 (17.06)	3190 (17.27)	1.45(0.91–2.31)	1.35 (0.81–2.27)	0.115	0.253

## Discussion

Previous studies concerned the seasonal and monthly variations of EGVB occurrence yielded conflicting results. It is mostly believed that EGVB had seasonal and monthly variations and tended to occur in cold season and months [8–11]. Fabrice Boulay et al. found that deaths (n = 13,514) and hospitalizations (n = 17,026) due to EGVB in France occurred with a clear annual periodicity and peaked in winter (December/January). While two studies in Spain and Japan did not show any monthly or seasonal fluctuations of EGVB occurrence [12]. Our study totally enrolled 4204 EGVB cases underwent emergency gastroscopic hemostasis in the Fifth Medical Center of PLA General Hospital in Beijing. Significant monthly and seasonal fluctuations (P < 0.0001 P < 0.0001) of the mean daily EGVB cases were found in this study, the mean daily EGVB cases increase obviously in the colder months (October to April) and winter. These results were consistent with most previous studies.

Low temperature exposure was thought to increase blood pressure, cardiac output, peripheral resistance and influence neuroendocrine factors and lead to a higher hepatic vein pressure gradient and cause EGVB [10, 17–20]. Three studies about the correlation between air temperature and EGVB occurrence also yielded conflicting conclusion [3, 12, 21]. In Nabil Tahri’s study, significant correlation was observed between mean temperature (p = 0003), rainfall (p < 0.01) and stormy weather (p = 0.008), while the mean temperature didn’t retain as an independent factor at multivariate analysis [21]. No significant relationship was found between EGVB and any of the climatic factors in a Spain study [12]. Wu et al*.* used a case cross-over study design and concluded that low air temperature increased the risk of EGVB as high as 14.4–30.7% per 5 °C decrease [3]. Then we further analyzed the association between the minimum temperature and emergency admission for EGVB using a time stratified case cross-over study design and conditional logistic regression methods. While the results were unexpected, no significant associations were observed between minimum temperature and risk of EGVB, and there was no significant difference when stratified by sex, age, etiology, liver cancer status, and grade of varices. These findings were not consistent with the previous study in Taiwan which also used a case cross-over study design and concluded that low air temperature increased the risk of EGVB regardless of age and sex [3]. The possible reasons might be involved: (1) Humoral agents and neurogenic factors might also influence the incidence of EGVB, seasonal changes in these substances might also be the causes of seasonal variations of emergency admission for EGVB [17, 18]. (2) Air pollution has been shown to be associated with the prevalence of many human diseases including gastrointestinal bleeding [22–24]. There was a large difference in ambient PM between the Beijing and Taiwan, that may lead to the difference in these two studies. (3) We couldn’t discriminate the indoor and outdoor temperature, indoor temperature was not in parallel with the outdoor due to indoor heating system in Beijing, this may lead to the different result. There might be other influencing factors for the monthly and seasonal fluctuations of EGVB occurrence in Beijing, and further study needed to be performed to explore the possible reasons and mechanisms.

There are some limitations in our study. First, most patients were first visit, we couldn’t get detailed information on the hepatic venous pressure gradient and Childs-Pugh score at baseline to further study the association between air temperature and EGVB in different disease stage. Secondly, not all patients requiring an emergency admission for EGVB in Beijing were included, while Fifth Medical Center of PLA General Hospital is the largest institution providing emergency gastroscopic hemostasis and a “Green Channel” of emergency medical service for EGVB patients in Beijing, and our study enrolled the majority of EGVB patients in Beijing. The last limitation was that we could not discriminate the indoor and outdoor air temperature; however, this is not highlighted as a major concern in most previous studies emphasizing the impact of air temperature on diseases.

## Conclusions

Emergency admission for EGVB showed significant monthly and seasonal fluctuations, while in conditional logistic regression analysis, no associations between air temperature and emergency admission for EGVB were observed.

## Data Availability

The datasets of this study are availability from the corresponding author on reasonable request.

## Abbreviations

EGVB: Esophagogastric variceal bleeding
EV: Esophagogastric varices
HCC: Hepatocellular carcinoma
RH%: Relative humidity
AP: Atmospheric pressure
DLNM: Distributed lag non-linear models
IQR: Interquartile range
